# Functional Group‐Driven Dissolution and Interfacial Instability in Naphthalene‐Derived Organic Anodes for Li‐Ion Storage

**DOI:** 10.1002/cssc.71197

**Published:** 2026-11-03

**Authors:** Michael Silvano, Nguyet N. T. Pham, Duc‐Anh Le, Yu‐Sheng Su

**Affiliations:** ^1^ International College of Semiconductor Technology National Yang Ming Chiao Tung University Hsinchu Taiwan, ROC; ^2^ Industry Academia Innovation School National Yang Ming Chiao Tung University Hsinchu Taiwan, ROC

**Keywords:** density functional theory, dissolution analysis, electrostatic potential maps, Li‐ion batteries, organic electrode materials, polycyclic aromatic hydrocarbons

## Abstract

The practical viability of small‐molecule organic anodes is often limited by active material dissolution and the accompanying interfacial instability in liquid electrolytes. Herein, we investigate how functional group chemistry influences Li/Li^+^ interactions, dissolution behavior, interfacial evolution, and electrochemical accessibility in naphthalene‐derived organic anodes. Using naphthalene as a model framework, we systematically compare hydroxylated and carboxylated derivatives through combined computational, electrochemical, spectroscopic, and morphological analyses. Density functional theory (DFT) calculations reveal stronger localized Li^+^ interactions at the vicinal dihydroxyl groups of 2,3‐dihydroxynaphthalene (DHN) and the carboxyl group of 2‐naphthoic acid (NPA) than in Naph and the monohydroxylated derivatives. However, this enhanced local affinity does not translate into improved electrochemical reversibility. Naphthalene and the monohydroxylated derivatives exhibit limited dissolution, relatively stable interfacial behavior, and sustained cycling performance over 1000 cycles. In contrast, DHN and NPA exhibit greater active material dissolution and more extensive interfacial reconstruction, resulting in reduced electrochemical accessibility and inferior reversibility. This work highlights the importance of balancing favorable Li/Li^+^ interactions with resistance to dissolution and interfacial reconstruction to preserve electrochemically accessible active material in small‐molecule organic anodes.

## Graphical abstract

Localized Li^+^ interactions at vicinal dihydroxyl and carboxyl groups are accompanied by greater dissolution and interfacial reconstruction in naphthalene‐derived organic anodes, whereas more delocalized interactions in naphthalene and monohydroxylated derivatives favor active‐material retention and more stable cycling.

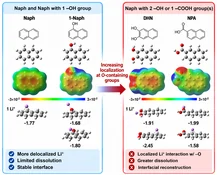

## 1 Introduction

Global decarbonization strategies, including net‐zero energy targets for 2050, rely heavily on the large‐scale deployment of lithium‐ion batteries (LIBs) for electric transportation and renewable energy storage [1]. This growing demand has intensified the search for electrode materials that combine high performance, long cycle life, low cost, and sustainability [2, 3]. While LIBs have gained widespread adoption due to their high energy density and operational reliability, the choice of electrode materials remains a critical determinant of cost, lifetime, and environmental impact [4].

Synthetic graphite currently dominates the commercial anode market owing to its stable capacity and mature manufacturing infrastructure [5]. However, graphitization typically requires temperatures above 2500 °C, leading to substantial energy consumption and carbon emissions [6]. These limitations have motivated the exploration of alternative anode materials that can deliver comparable electrochemical performance while enabling more energy‐efficient and sustainable processing routes. Among the emerging candidates, organic electrode materials have attracted increasing attention due to their structural tunability, potential sustainability, and compatibility with low‐temperature synthesis [7, 8]. Over the past several decades, a broad range of redox‐active organic compounds, including carbonyl compounds, carboxylates, organic radicals, organosulfur species, and conjugated molecular systems, have been investigated for rechargeable batteries, establishing molecular design as a viable route for tailoring redox potential, ion‐storage chemistry, and electrode functionality [7, 8, 9, 10, 11]. Despite these advantages, organic electrodes commonly face limitations associated with dissolution in liquid electrolytes, low intrinsic electronic conductivity, and low volumetric density, making molecular structure and electrolyte compatibility central considerations in their design [9, 10, 11].

For organic anodes specifically, early studies demonstrated reversible low‐potential lithium storage in conjugated dicarboxylates, establishing that appropriately designed organic molecules can operate as alternatives to conventional inorganic insertion hosts [12]. Aromatic π‐systems have also been investigated as Li‐storage motifs; studies on fused C_6_ aromatic frameworks demonstrated that lithiation can extend beyond conventional intercalation concepts and involve direct interaction with the conjugated aromatic framework [13]. More recently, small‐molecule polycyclic aromatic hydrocarbons (PAHs), including naphthalene, biphenyl, 9,9‐dimethylfluorene, phenanthrene, and related compounds, have been shown to support low‐potential Li storage with long‐term cycling stability, while their performance is strongly influenced by the molecular structure and dissolution behavior [14, 15].

Molecular functionalization is one of the principal strategies for tuning the electrochemical properties of organic electrode materials. Changes in functional group chemistry can modify redox energetics, electronic structure, ion coordination, molecular packing, and solubility, and these effects have motivated extensive molecular engineering approaches for organic batteries [16, 17, 18, 19, 20, 21]. For small organic molecules, however, structural modification can introduce competing effects. Enhancing ion affinity or modifying the molecular electronic structure does not necessarily improve cycling stability because the same substitution can also alter solubility and electrode–electrolyte interactions. Dissolution remains a particularly important limitation for low‐molecular‐weight organic electrodes in conventional liquid electrolytes [20, 22]. Within a common aromatic framework, what is less clear is how specific changes in the position, number, and chemical identity of oxygen‐containing substituents alter Li/Li^+^ interactions while simultaneously affecting dissolution and interfacial evolution. Distinguishing these coupled effects is particularly important for small‐molecule PAH anodes, where the electrochemically active π‐framework and the electrolyte‐accessible molecular structure are intrinsically linked.

In this work, we systematically investigate the effect of functional group chemistry on lithium storage in naphthalene‐derived small molecules. Using naphthalene (Naph) as a model framework, we compare its electrochemical behavior with that of hydroxylated and carboxylated derivatives, including 2,3‐dihydroxynaphthalene (DHN), 1‐naphthol (1‐Naph), 2‐naphthol (2‐Naph), and 2‐naphthoic acid (NPA). This molecular series enables three related structural comparisons: 1‐Naph and 2‐Naph probe the effect of hydroxyl substitution position; comparison with DHN examines the influence of increasing hydroxyl functionality; and NPA provides a carboxyl‐containing comparator for distinguishing oxygen content from functional‐group identity. These comparisons allow the effects of oxygen‐containing substituents on Li/Li^+^ interaction, dissolution behavior, interfacial evolution, and electrochemical accessibility to be examined within a common naphthalene framework. By integrating density functional theory (DFT)‐derived electrostatic characteristics and Li/Li^+^ adsorption behavior with electrochemical, spectroscopic, and morphological analyses, this study reveals a coupled mechanism in which functional‐group‐induced dissolution and interphase reconstruction jointly govern lithium‐ion storage performance and provides design guidelines for the development of stable small‐molecule organic anodes.

## 2 Experimental Section

### 2.1 Materials and Electrode Fabrication

Analytical‐grade naphthalene and its derivatives were used as active materials, including naphthalene (99%, ACROS), 2,3‐dihydroxynaphthalene (98%, Thermo Scientific), 1‐naphthol (99%, Thermo Scientific), 2‐naphthol (98+%, Thermo Scientific), and 2‐naphthoic acid (98+%, Thermo Scientific). Working electrodes were prepared by casting an aqueous slurry composed of active material (70 wt%), Super P conductive carbon black (20 wt%, Imerys), carboxymethyl cellulose (CMC, 5 wt%, Dow), and styrene–butadiene rubber (SBR, 5 wt%, JSR). The slurry was homogenized by mechanical stirring and coated onto a precleaned battery‐grade copper foil current collector. To mitigate sublimation or melting of the active materials (Table 1), a mild drying protocol was adopted. The coated electrodes were vacuum‐dried at 40 °C for 24 h. After drying, the electrodes were calendered to improve mechanical integrity and punched into disks with a diameter of 1.5 cm.

**TABLE 1:** Physical properties of naphthalene‐based PAHs.

PAHs	Molar mass, g mol^−1^	Density, g cm^−3^	Melting point, °C	Boiling point, °C
Naphthalene	128.17	1.16	79–82	218
2,3‐Dihydroxynaphthalene	160.17	1.34	162–165	354
1‐Naphthol	144.17	1.28	95	278
2‐Naphthol	144.17	1.28	120	285
2‐Naphthoic acid	172.18	1.08	182	>300

### 2.2 Cell Assembly and Electrochemical Measurements

CR2032‐coin‐type half‐cells were assembled using the prepared electrodes as working electrodes and lithium foil as the counter/reference electrode, separated by a Celgard 2400 membrane. The electrolyte consisted of 1.0 M LiPF_6_ dissolved in a binary solvent mixture of ethylene carbonate and ethyl methyl carbonate (EC/EMC, 1:2 v/v). Cell assembly was carried out in an argon‐filled glovebox with O_2_ and H_2_O levels below 0.5 ppm. Galvanostatic charge–discharge (GCD) measurements were performed using a Neware CT4008 battery tester. The first lithiation was recorded from the OCV to 0.01 V, and subsequent charge–discharge cycling was conducted between 0.01 and 1.5 V versus Li/Li^+^. Current densities were expressed in C‐rate, where 1 C corresponds to a full charge or discharge within 1 h. The specific capacities of the naphthalene‐derived electrodes were calculated based on the mass of the molecular active material. Cyclic voltammetry (CV) measurements were conducted using a PalmSens EmStat4 potentiostat. Electrochemical impedance spectroscopy (EIS) was performed using a BioLogic SP‐50e system over a frequency range from 1 MHz to 50 mHz.

### 2.3 Materials Characterization

The morphology of pristine and cycled electrodes was examined by scanning electron microscopy (SEM, Hitachi SU‐8010). Fourier transform infrared (FTIR) spectra were recorded using a Bruker Vertex 80 v spectrometer equipped with a KBr beam splitter and a DTGS detector. Measurements were conducted in the absorbance mode with a resolution of 2 cm^−1^ over 200 scans. Surface chemical composition was analyzed by X‐ray photoelectron spectroscopy (XPS, ULVAC‐PHI Quantera II) with Ar^+^ sputtering for depth profiling. To evaluate dissolution behavior, electrolyte solutions collected after static soaking of the molecular compounds and from cycled cells were analyzed by ultraviolet–visible spectroscopy (UV–vis, Jasco V‐730).

### 2.4 Computational Methods

DFT calculations were performed using the Gaussian 16 package [23]. The molecular geometries of Naph, DHN, 1‐Naph, 2‐Naph, and NPA were fully optimized using the ωB97XD functional with the 6‐311+G(d,p) basis set. Electrostatic potential (ESP) maps were generated from the optimized molecular structures to examine the spatial distribution of ESP. To evaluate Li/Li^+^ interactions, various adsorption configurations were considered for each molecule, including aromatic‐ring‐centered and oxygen‐containing sites, where applicable. The adsorption energy was calculated as:

(1) $$E_{\mathrm{ads}}\;=\;E_{\mathrm{tot}}\;-\;E_{\mathrm{PAH}}\;-\;E_{\mathrm{Li}/{\mathrm{Li}}^{+}}$$

where the $E_{\mathrm{tot}}$, the $E_{\mathrm{PAH}}$, and the $E_{\mathrm{Li}/{\mathrm{Li}}^{+}}$ represent the total energies of the optimized adsorbed system, isolated molecule, and isolated Li atom or Li^+^ ion, respectively. A more negative adsorption energy indicates a more energetically favorable interaction.

## 3 Results and Discussion

### 3.1 Electrochemical Response and Initial Irreversibility

To elucidate how molecular functionalization influences the electrochemical response of a common aromatic framework, the initial GCD behavior of Naph, DHN, 1‐Naph, 2‐Naph, and NPA was systematically examined (Figure 1a). These compounds share a common fused naphthalene backbone but differ in hydroxyl position, hydroxyl number, and oxygen‐containing functional‐group identity. As shown in Figure 1b–f, all electrodes exhibit discernible electrochemical activity during the first discharge–charge cycle, confirming that the naphthalene core remains electrochemically accessible after functionalization. However, substantial differences are observed in the voltage profiles, including variations in sloping versus plateau‐like regions and the degree of voltage hysteresis, indicating that the lithiation/delithiation processes are strongly dependent on the functional group. The initial cathodic features observed in the range of ~0.6–0.9 V can be attributed primarily to electrolyte decomposition and the formation of a solid electrolyte interphase (SEI) [16], suggesting that interfacial reactions contribute significantly to the first‐cycle electrochemical response.

**FIGURE 1:**
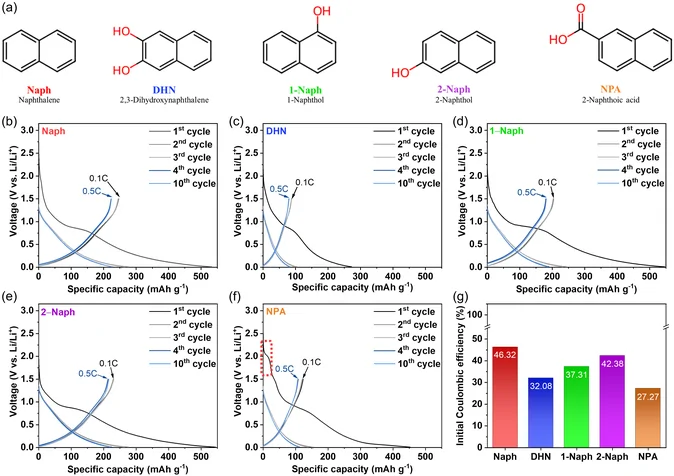
Electrochemical response of naphthalene‐derived organic electrodes during the initial cycles. (a) Molecular structures of Naph, DHN, 1‐Naph, 2‐Naph, and NPA, representing variations in hydroxyl position, hydroxyl number, and oxygen‐containing functional‐group identity. (b–f) GCD profiles of the corresponding electrodes during the initial cycles. (g) Comparison of ICEs. All electrodes exhibit electrochemical activity associated with the naphthalene framework, while distinct variations in voltage profiles and ICE values indicate that functional‐group substitution strongly influences initial lithiation behavior and the extent of irreversible electrochemical processes.

Quantitatively, the first‐discharge capacities of Naph, DHN, 1‐Naph, 2‐Naph, and NPA are 533, 273, 547, 544, and 453 mAh g^−1^, respectively, while the corresponding first‐charge capacities are 247, 88, 204, 231, and 123 mAh g^−1^, leading to initial Coulombic efficiencies (ICEs) of 46.32%, 32.08%, 37.31%, 42.38%, and 27.27% (Figure 1g). To assess the electrochemical background from the nonactive electrode components, an active material‐free Super P/CMC/SBR electrode was tested under identical conditions (Figure S1). The control electrode exhibits only a low reversible capacity (15 mAh g^−1^ at 0.5 C), indicating that the conductive carbon/binder background contributes only a minor fraction of the capacities measured for the molecular electrodes. The pronounced irreversibility observed across all samples indicates that a significant fraction of the initial electrochemical process is consumed by parasitic reactions, including SEI formation and possible side reactions associated with active material instability. For the hydroxylated derivatives, an additional possible contribution is the chemical evolution of phenolic O─H groups under deep lithiation. At potentials approaching 0.01 V versus Li/Li^+^, partial deprotonation and/or formation of O─Li‐containing species cannot be excluded and may contribute to irreversible charge consumption and interphase formation during the initial cycle. Compared to Naph and the monohydroxylated derivatives (1‐Naph and 2‐Naph), DHN and NPA exhibit lower ICE values and more distorted voltage profiles, indicating less efficient electrochemical utilization during the first cycle. NPA even possesses an additional irreversible plateau at ~2.0 V during the first lithiation process (Figure 1f). This behavior suggests that increasing oxygenated functionality does not necessarily enhance lithium‐ion storage reversibility; instead, it may introduce additional interfacial reactivity and alter the Li/Li^+^ interaction environment. In particular, the significantly reduced ICE observed for DHN and NPA is consistent with more extensive irreversible electrochemical processes, whereas the relatively higher ICE of Naph and 2‐Naph reflects a more favorable balance between reversible charge storage and parasitic consumption. These results indicate that functional group substitution not only affects the magnitude of stored charge but also fundamentally modifies the electrochemical pathways governing initial lithiation.

### 3.2 Molecular Electrostatics and Li/Li^+^ Adsorption

To gain molecular‐level insight into the effects of oxygen functionalization on Li/Li^+^ interactions, the optimized geometries and ESP distributions of Naph, DHN, 1‐Naph, 2‐Naph, and NPA were examined (Figure 2a). The ESP maps reveal a pronounced redistribution of the negative ESP upon oxygen functionalization. For Naph, the negative potential is primarily distributed near the centers of the aromatic rings. In 1‐Naph and 2‐Naph, the negative potential becomes partially localized around the hydroxyl O atom, whereas DHN exhibits strongly negative regions around both hydroxyl groups. NPA shows a pronounced negative potential near the carboxyl O atoms, particularly the carbonyl O. Consistent with the ESP maps, the calculated dipole moments are 0.0005, 0.85, 1.53, 1.63, and 2.12 D for Naph, DHN, 1‐Naph, 2‐Naph, and NPA, respectively, reflecting the redistribution of molecular polarity upon oxygen functionalization. These results demonstrate that oxygen‐containing substituents substantially modify the local electrostatic environment of the naphthalene framework.

**FIGURE 2:**
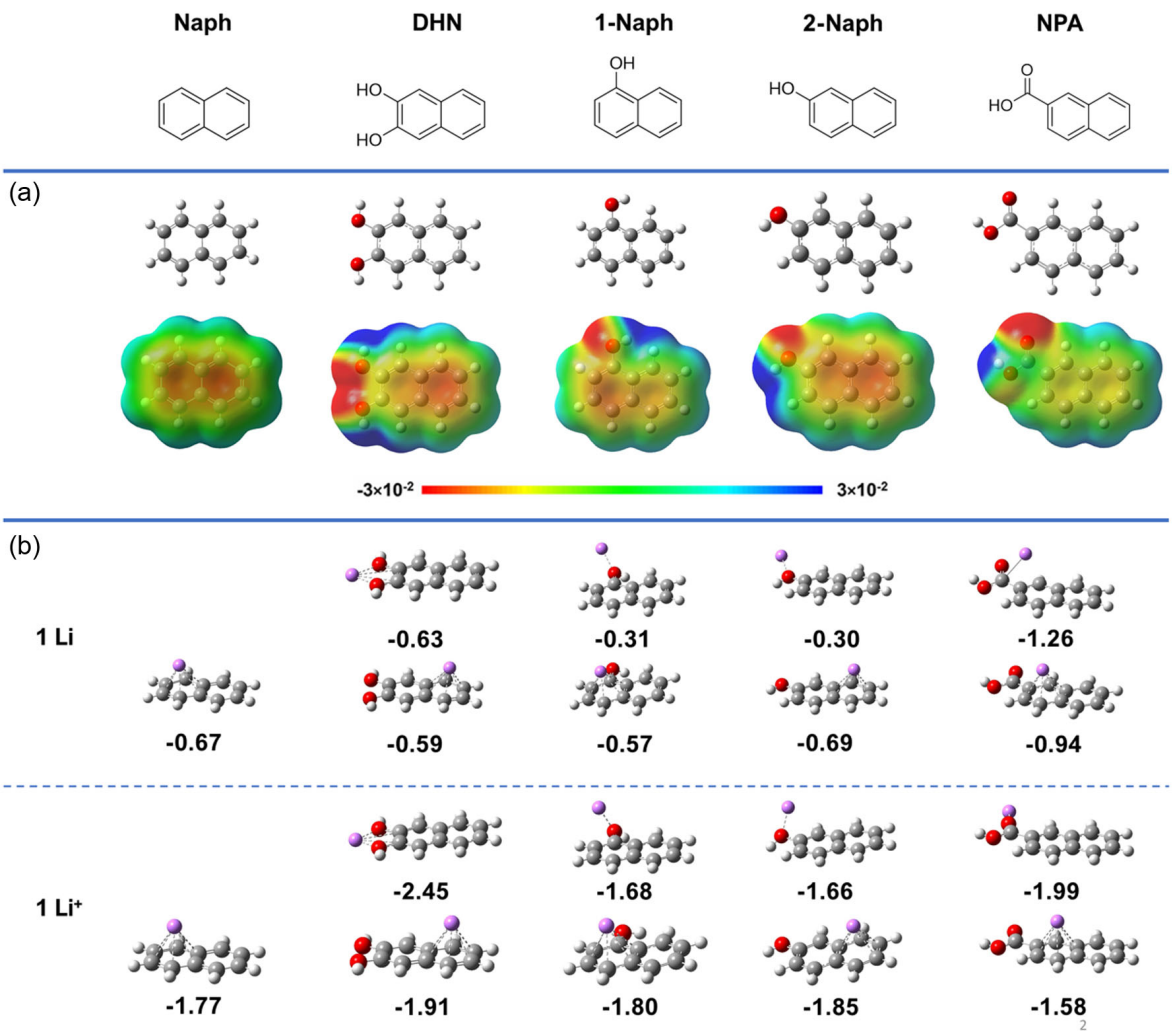
Molecular electrostatic potentials and Li/Li^+^ adsorption on naphthalene derivatives. (a) Optimized molecular structures and electrostatic potential (ESP) mapped onto an electron‐density isosurface of 0.0004 |e|/Bohr^3^. (b) Optimized Li and Li^+^ adsorption configurations at representative aromatic‐ring and oxygen‐containing sites of Naph, DHN, 1‐Naph, 2‐Naph, and NPA, with the corresponding adsorption energies $E_{\mathrm{ads}}$.

The Li and Li^+^ adsorption behaviors were further examined at representative aromatic‐ring and oxygen‐containing sites (Figure 2b). For Naph, Li and Li^+^ preferentially adsorb near the aromatic‐ring center, with adsorption energies of −0.67 and −1.77 eV, respectively. A similar ring‐centered preference is retained for the monohydroxylated derivatives. For 1‐Naph, the Li^+^ adsorption energy at the aromatic ring is −1.80 eV, compared with −1.68 eV near the hydroxyl group, whereas the corresponding values for 2‐Naph are −1.85 and −1.66 eV. In contrast, DHN shows a clear preference for the hydroxyl‐containing region, particularly for Li^+^, with an adsorption energy of −2.45 eV compared with −1.91 eV at the aromatic ring. For NPA, the carboxyl group also provides the preferred adsorption site, with Li and Li^+^ adsorption energies of −1.26 and −1.99 eV, respectively, compared with −0.94 and −1.58 eV at the aromatic ring. These site preferences indicate only a modest effect of the hydroxyl position in the monohydroxylated isomers, whereas increasing hydroxyl functionality in DHN or introducing a carboxyl group in NPA shifts the preferred Li/Li^+^ interaction toward oxygen‐containing regions. Thus, both the number and chemical identity of oxygen‐containing groups strongly influence the local Li/Li^+^ interaction environment.

Comparison with the first‐cycle electrochemical response shows that stronger Li/Li^+^ adsorption does not directly translate into improved reversibility, as DHN and NPA exhibit relatively strong Li^+^ adsorption but lower ICEs and reversible capacities than Naph and the monohydroxylated derivatives (Figure 1). The apparent reversible Li uptake estimated from the first‐charge capacities is approximately 1.18, 0.53, 1.10, 1.24, and 0.79 Li per molecular unit for Naph, DHN, 1‐Naph, 2‐Naph, and NPA, respectively. These values should be regarded as apparent electrochemical equivalents rather than discrete lithiation stoichiometries. Thus, local Li/Li^+^ affinity alone does not determine electrochemical reversibility, and dissolution and interfacial evolution must also be considered.

### 3.3 Cycling Performance and Dissolution‐Associated Capacity Loss

To further evaluate the electrochemical evolution beyond the initial cycle, the cycling behavior of the naphthalene‐derived electrodes was examined through GCD profiles at selected cycle numbers, together with long‐term cycling performance (Figure 3a–f). Following the initial activation stage, all electrodes remain electrochemically active, and the Coulombic efficiency rapidly approaches ~100%, indicating that the dominant irreversible processes are largely confined to the first cycle and that a relatively stable electrochemical regime is subsequently established. Despite this apparent stabilization, the electrodes converge to markedly different reversible capacities. Naph retains the highest capacity, while 1‐Naph and 2‐Naph stabilize at intermediate levels, whereas DHN and especially NPA exhibit substantially lower stabilized capacities. The corresponding capacity retentions after prolonged cycling are 104.83%, 93.63%, 96.67%, 92.04%, and 88.11% for Naph, DHN, 1‐Naph, 2‐Naph, and NPA, respectively. The slight increase in capacity retention for Naph is likely associated with gradual electrode activation during early cycling. Importantly, this divergence is established during early cycles rather than arising from continuous long‐term fading, suggesting that the accessible electrochemical capacity is primarily determined by processes occurring during the initial electrochemical adaptation stage. The evolution of the GCD profiles further supports this interpretation. For Naph, 1‐Naph, and 2‐Naph, the charge–discharge curves progressively evolve into more reproducible shapes with cycling, consistent with the establishment of stable lithiation/delithiation pathways. These observations suggest that the inferior performance of DHN and NPA arises primarily from the early loss of electrochemically accessible material rather than continuous long‐term degradation.

**FIGURE 3:**
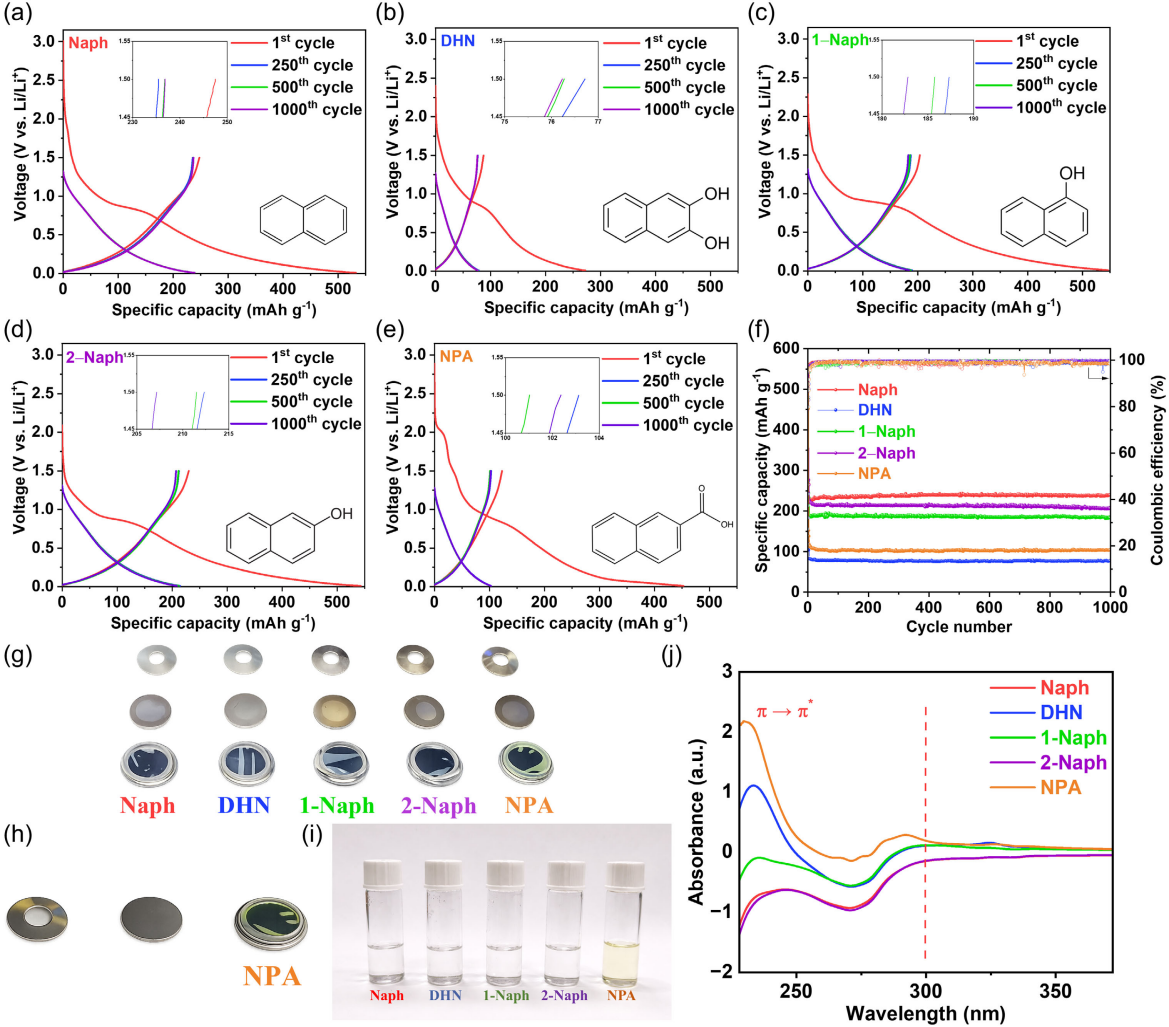
Cycling evolution and dissolution behavior of naphthalene‐derived electrodes. (a–e) GCD profiles at selected cycles. (f) Long‐term cycling performance and corresponding Coulombic efficiency. (g) Optical images of disassembled cells after cycling. (h) Enlarged view of the NPA electrode after cycling, showing pronounced discoloration and residue formation. (i) Photograph of the recovered electrolytes. (j) UV–vis spectra of corresponding electrolytes.

Additional qualitative evidence is provided by the postcycling optical images (Figure 3g,h). Compared with the other electrodes, the NPA cell exhibits pronounced discoloration and visible residue on the separator and electrode surfaces after cycling. Such features indicate extensive electrolyte‐side reactions and the migration of soluble species away from the electrode. In contrast, Naph, 1‐Naph, and 2‐Naph show comparatively cleaner surfaces with minimal visual change, suggesting better retention of the active material within the electrode framework, while DHN exhibits intermediate behavior. These macroscopic observations are consistent with the electrochemical trends and provide direct visual evidence of dissolution‐associated processes.

To determine the origin of the capacity divergence, the extent of active material dissolution was evaluated by analyzing the recovered electrolytes after cycling (Figure 3i,j). The electrolyte from the NPA cell exhibits a pronounced yellow coloration, whereas those from the other electrodes remain relatively clear. The corresponding UV–vis spectra reveal a significantly stronger absorbance for NPA, indicating a stronger dissolution‐related optical response. DHN also shows moderate absorbance, while Naph, 1‐Naph, and 2‐Naph display only weak signals. To determine whether this trend originates solely from electrochemical cycling, the five molecular compounds were also statically soaked in the same electrolyte for 1 and 10 days without electrochemical cycling (Figure S2). Naph retains only weak UV–vis absorption at both soaking times, whereas the oxygen‐functionalized derivatives exhibit stronger but material‐dependent responses. The persistence of weak dissolution for Naph in the absence of electrochemical reactions indicates that its limited dissolution is not a consequence of redox reactions.

The comparatively limited dissolution observed for Naph can be rationalized by its distinct molecular electrostatic character. As shown by the DFT‐derived ESP maps, Naph lacks localized oxygen‐centered negative electrostatic regions, and its negative potential is instead distributed across the aromatic π‐framework. In contrast, oxygen functionalization generates more localized negative electrostatic regions around the hydroxyl or carboxyl groups. The different dissolution responses among the functionalized derivatives suggest that both the number and chemical identity of the oxygen‐containing groups influence dissolution. Thus, the more delocalized electrostatic character of Naph provides a plausible molecular‐level rationale for its comparatively limited dissolution under the present electrolyte conditions. These results are consistent with the mass change data (Table S1), which support a greater net material loss for NPA and DHN during cycling. The reduced reversible capacities of these two electrodes are therefore closely associated with the depletion of electrochemically accessible active material. The stronger localized Li^+^ interactions predicted at the oxygen‐containing functional groups of DHN and NPA further suggest that lithiation is concentrated around these substituents, which may induce greater perturbation of the local molecular environment and contribute to active‐material loss during cycling. As a result, a significant fraction of active material becomes electrochemically inaccessible after the initial cycles, leading to the lower stabilized capacities.

### 3.4 Structural Reconstruction and Kinetic Behavior

To understand how dissolution‐induced material loss influences electrochemical behavior, the structural evolution and kinetic characteristics of the electrodes were examined by SEM and electrochemical measurements. The morphology of the electrodes before and after cycling is shown in Figure 4a–j. All pristine electrodes exhibit a porous granular network, typical of composite electrodes consisting of active material, conductive carbon, and binder. However, structural differences are already evident between the materials. In particular, the DHN electrode contains larger plate‐like domains in addition to the granular framework (Figure 4b), which can be attributed to the intrinsic morphology of the DHN powder (Figure 4k–m). After cycling, all electrodes underwent varying degrees of structural reconstruction. Naph and the monohydroxylated derivatives (1‐Naph and 2‐Naph) retain a relatively discernible granular morphology, although partial densification is observed. In contrast, the plate‐like domains observed in pristine DHN are no longer discernible (Figure 4g), and NPA exhibits significantly more compact and coalesced structures (Figure 4j), indicating substantial restructuring of these electrode surfaces after cycling. This densification is consistent with the loss of active material into the electrolyte, as established in Figure 3j, together with the accumulation of interphase‐derived products on the residual electrode framework.

**FIGURE 4:**
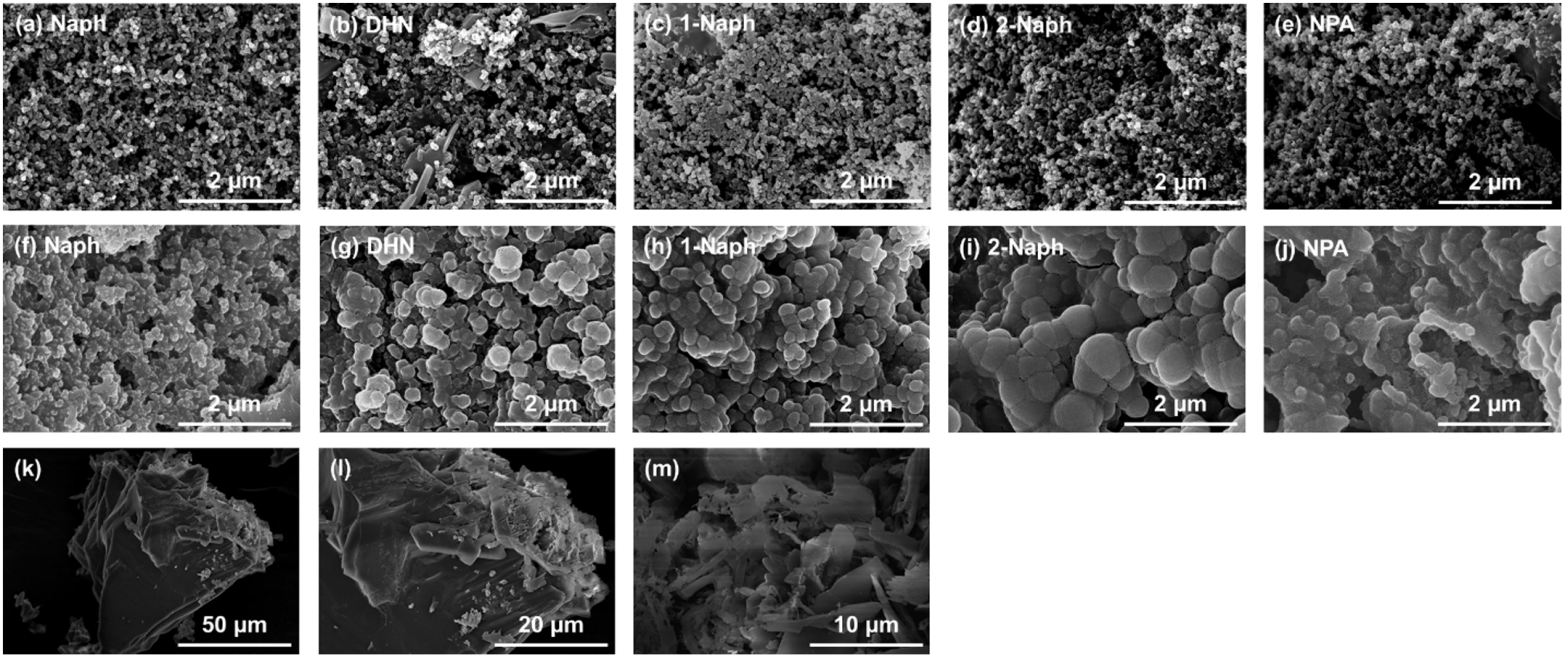
Structural reconstruction of naphthalene‐derived electrodes before and after cycling. (a–e) SEM images of pristine electrodes (Naph, DHN, 1‐Naph, 2‐Naph, and NPA). (f–j) Corresponding SEM images after cycling. (k–m) Pristine DHN powder morphology at different magnifications.

From an electrochemical perspective, such structural evolution is expected to influence ion/electron transport and the accessibility of active sites. To evaluate this effect, the rate performance of the electrodes was examined at different current densities (Figure 5a–f). All electrodes show decreasing capacity with increasing current density, reflecting increasing kinetic limitations. However, the extent of capacity retention differs significantly among the materials. Naph exhibits the most favorable rate capability, while 1‐Naph and 2‐Naph show moderate performance. In contrast, both DHN and NPA display substantially reduced capacities under identical conditions, indicating limited electrochemical accessibility under nonequilibrium operation, which can be attributed to the combined effects of active material depletion and interfacial reconstruction.

**FIGURE 5:**
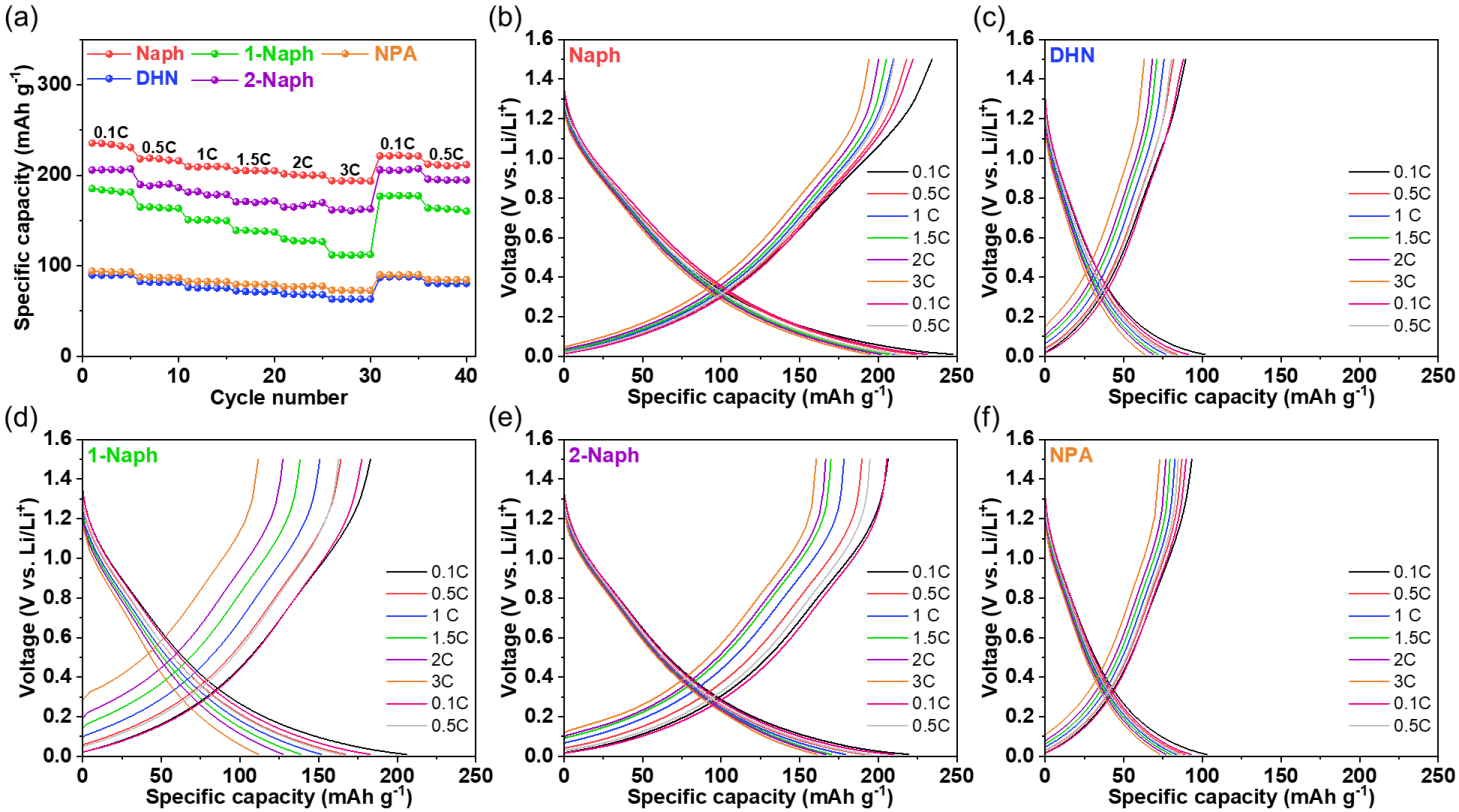
Rate capability and corresponding electrochemical response of naphthalene‐derived electrodes. (a) Reversible capacities at sequential current densities. (b–f) GCD profiles at different current densities.

Further insight into the charge storage mechanism was obtained from CV. During the initial cathodic scan, all electrodes exhibit reduction features associated with SEI formation in the range of ~0.6–0.7 V, followed by low‐potential redox processes below ~0.2 V corresponding to lithium storage within the molecular framework (Figure 6a–e) [8, 14, 15, 24]. Notably, DHN and especially NPA exhibit broader and earlier cathodic responses with less current output during deep lithiation, indicating stronger interfacial reactivity and more extensive irreversible processes during the first cycle, consistent with their lower ICEs. The CV curves become more stable in subsequent cycles, indicating that the dominant interfacial reactions occur during the first cycle and that a quasistable electrochemical environment is established thereafter. The charge‐storage kinetics were further analyzed using the power‐law relationship and current separation method:

(2) $$i\;=\;av^{b}$$

(3) logi = loga + blogv

(4) $$i(V)\;=\;k_{1}v\;+\;k_{2}v^{1/2}$$

**FIGURE 6:**
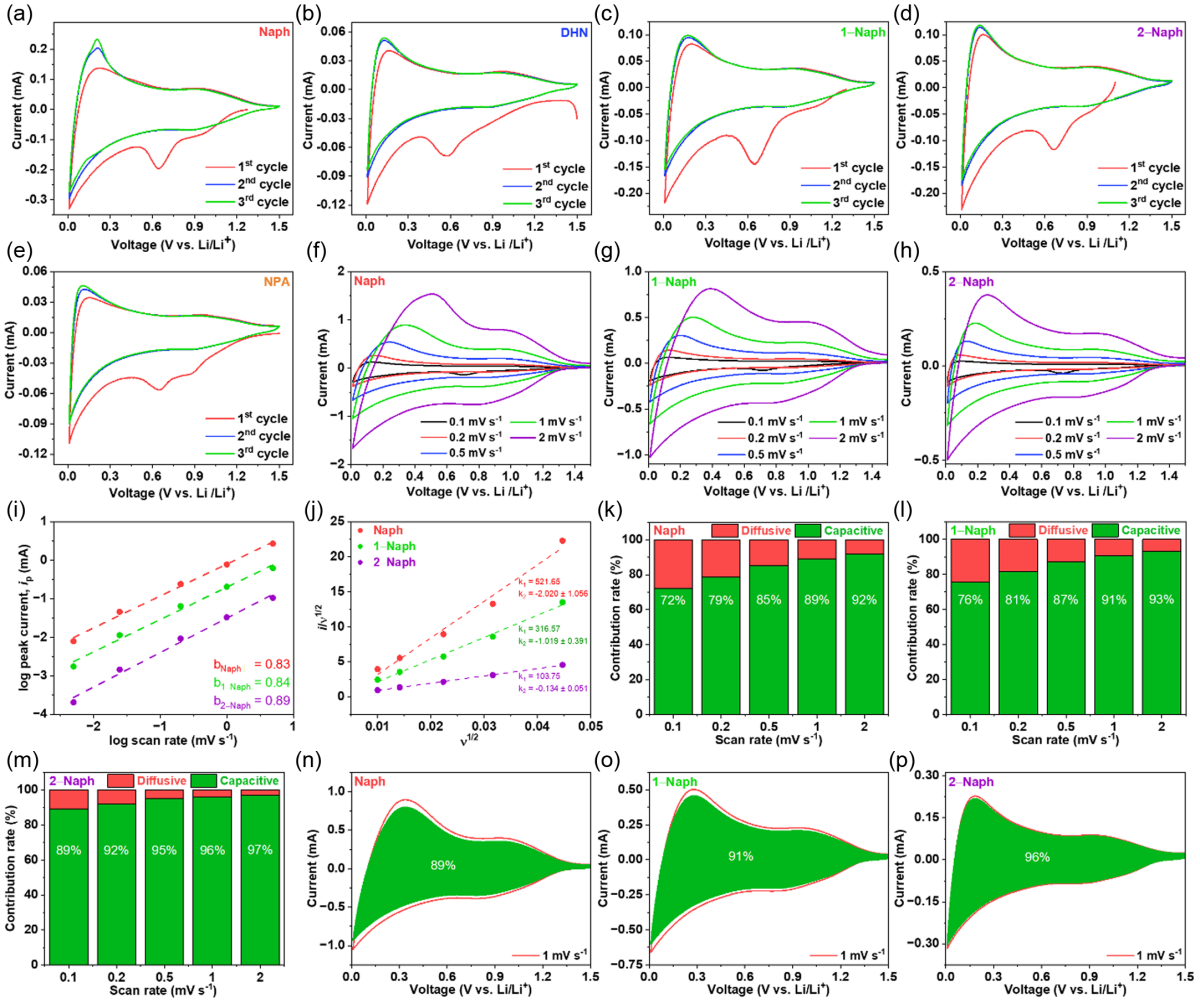
CV analysis of the charge storage behavior in naphthalene‐derived electrodes. (a–e) CV curves at 0.2 mV s^−1^. (f–h) CV curves at varying scan rates. (i, j) Plots for *b*‐value and *k*_1_/*k*_2_ value determination. (k–m) Capacitive contribution as a function of scan rate. (n–p) Representative separation of capacitive and diffusion‐controlled current at 1 mV s^−1^.

where i is the current, v is the scan rate, b reflects the charge‐storage mechanism, and $k_{1}$ and $k_{2}$ correspond to capacitive and diffusion‐controlled contributions, respectively [14, 25]. Quantitative analysis yields *b*‐values of 0.83, 0.84, and 0.89 for Naph, 1‐Naph, and 2‐Naph, respectively, consistent with predominantly capacitive kinetics (Figure 6f–j) [14, 25]. The capacitive contribution further increases with scan rate and exceeds 90% at higher scan rates (Figure 6k–p). Combined with the DFT results, the readily accessible ring‐centered Li/Li^+^ interaction sites in these molecules are consistent with their predominantly capacitive response and favorable rate capability, while better active material retention supports stable charge‐storage kinetics.

### 3.5 Interfacial Evolution and Surface Chemistry

To clarify how molecular functionalization influences the electrode/electrolyte interface during cycling, EIS, FTIR, and XPS analyses were carried out to probe both the evolution of interfacial resistance and the chemical nature of the surface species formed after cycling. The EIS results show that all electrodes undergo substantial interfacial evolution during the initial cycle, as evidenced by the pronounced changes in the Nyquist plots and fitted resistance parameters (Figure 7) [14, 15]. This behavior indicates that rapid restructuring of the electrode/electrolyte interface takes place during early lithiation/delithiation. However, the subsequent interfacial evolution differs markedly depending on the functional group. Among all derivatives, Naph exhibits the most favorable impedance behavior (Table S2). It shows a relatively low initial charge‐transfer resistance before cycling (*R*_ct_ = 84.1 Ω) and after prolonged cycling still maintains comparatively low SEI and charge‐transfer resistances (*R*_SEI_ = 9.7 Ω and *R*_ct_ = 33.2 Ω at the 100th cycle), indicating the formation of a relatively stable interphase that does not severely impede subsequent lithium‐ion storage reactions. A similar trend is observed for 1‐Naph, which also retains relatively small long‐term resistance values (*R*_SEI_ = 6.3 Ω and *R*_ct_ = 60.1 Ω at the 100th cycle), consistent with its good reversible capacity and kinetically accessible charge storage behavior. In contrast, DHN and especially NPA exhibit much less favorable interfacial characteristics. DHN develops substantial interphase‐related resistance during the first cycle (*R*_SEI_ = 255.2 Ω and *R*_ct_ = 524.3 Ω), and although its *R*_ct_ decreases significantly by the 100th cycle (31.0 Ω), the persistently high *R*_SEI_ (180.8 Ω) indicates that the electrode surface remains covered by a highly resistive interphase. NPA shows the most severe impedance growth, with both *R*_SEI_ and *R*_ct_ increasing markedly upon continued cycling (*R*_SEI_ = 281.9 Ω and *R*_ct_ = 510.3 Ω at the 100th cycle), indicating pronounced interfacial resistance growth that strongly hinders charge transfer. These EIS results are consistent with the reduced electrochemical accessibility and electrode instability observed for DHN and NPA. Comparison with the DFT results in Figure 2 further shows that the impedance trend does not simply follow Li/Li^+^ adsorption strength. DHN and NPA exhibit stronger localized Li^+^ interactions yet higher long‐term interfacial resistance than Naph. Thus, Li/Li^+^ affinity alone does not determine interfacial stability; active material dissolution and the resulting interphase evolution must also be considered.

**FIGURE 7:**
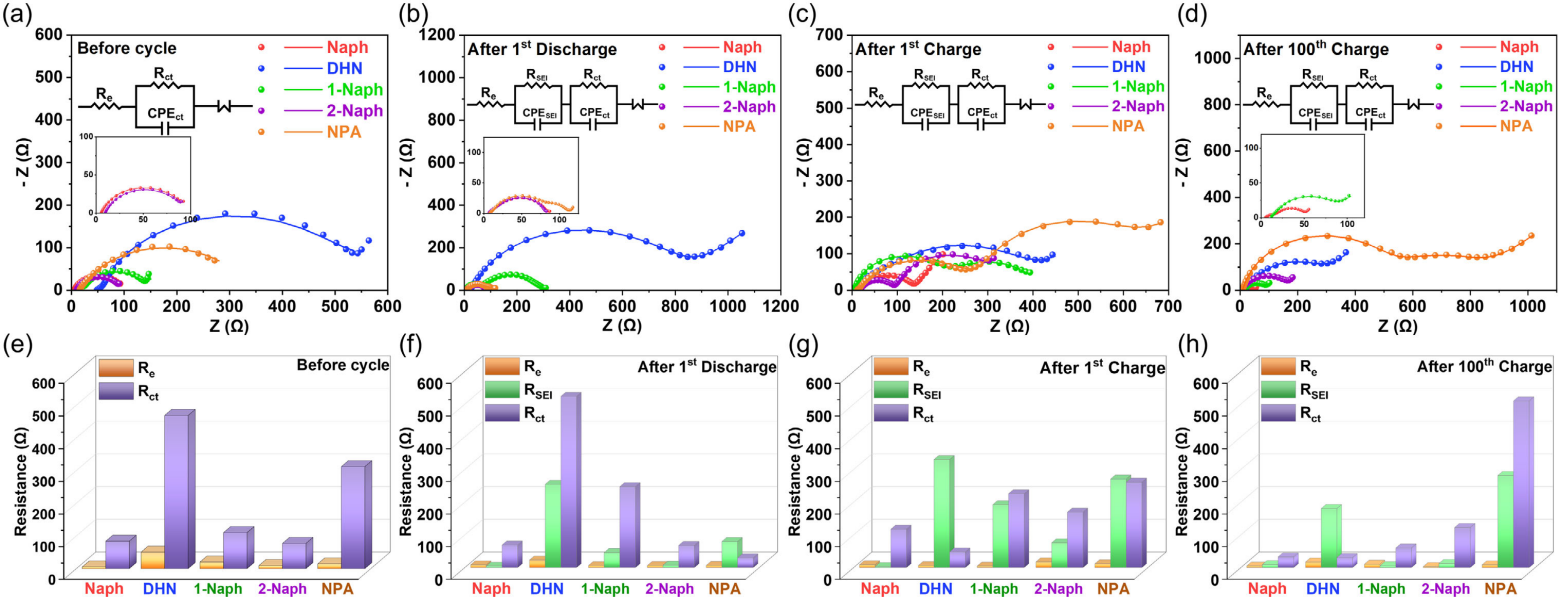
Interfacial evolution of naphthalene‐derived electrodes analyzed by EIS. (a–d) Equivalent circuits and Nyquist plots at different cycling states. (e–h) Fitted resistance parameters (*R*_e_, *R*_SEI_, and *R*_ct_) derived from the impedance spectra.

To obtain chemical insight into this interfacial evolution, FTIR spectra were collected for the pristine, first‐lithiated, and first‐delithiated electrodes (Figure 8a–f). The pristine electrodes largely retain the characteristic vibrational features of the parent molecules. In general, the spectra contain bands associated with O─H, C─H stretching, aromatic C═C, C─O, and aromatic C─H out‐of‐plane bending, while NPA additionally shows distinct C═O and C═C/COO^−^ contributions arising from its carboxylic functionality [26, 27, 28]. Clear spectral evolution occurs during the first lithiation/delithiation cycle. For the hydroxylated derivatives, the O─H‐related vibrations decrease after the first lithiation and partially recover upon delithiation, indicating a substantial and partially reversible change in the local hydroxyl environment. This behavior is consistent with strong interaction of the phenolic oxygen‐containing groups with Li during deep lithiation. However, the FTIR data alone cannot distinguish among Li coordination, partial deprotonation, and other interfacial reactions involving the phenolic groups. Importantly, the recovery of the O─H‐related features after delithiation is accompanied by enhanced C─O and COO^−^‐region features, indicating that the delithiated surface does not simply return to the pristine molecular state but retains additional oxygen‐containing species formed during the initial electrochemical cycle. The appearance of an O─H‐related feature for Naph after delithiation further suggests that part of the broad O─H response may originate from electrolyte‐derived interphase species rather than exclusively from intrinsic phenolic O─H groups.

**FIGURE 8:**
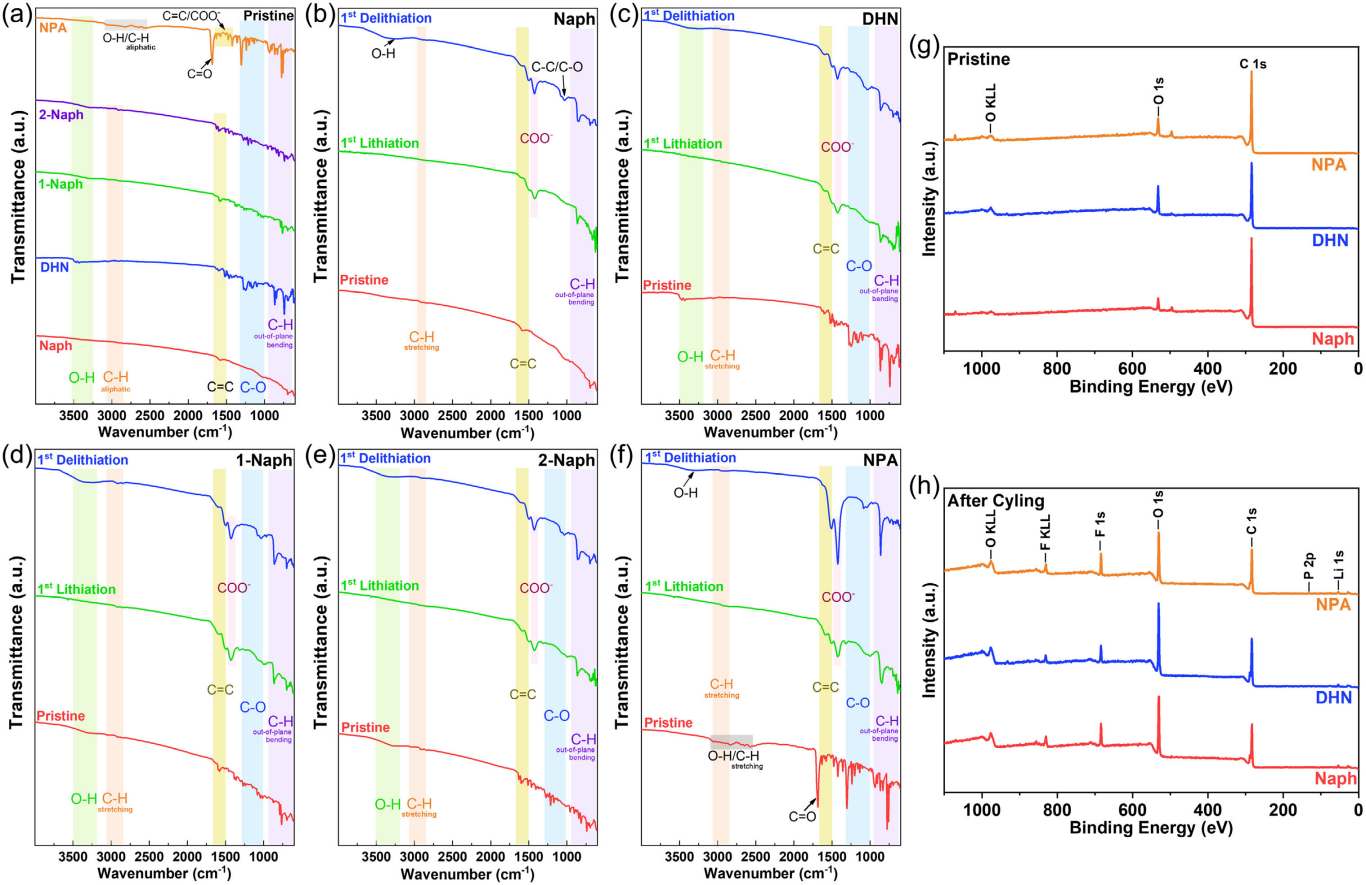
Surface chemical evolution of representative naphthalene‐derived electrodes. (a–f) FTIR spectra of the pristine, first‐lithiated, and first‐delithiated Naph, DHN, 1‐Naph, 2‐Naph, and NPA electrodes. (g, h) XPS survey spectra of Naph, DHN, and NPA electrodes before and after cycling.

The extent of this chemical evolution remains material dependent. DHN exhibits more pronounced spectral evolution, including stronger changes in the oxygen‐containing and aromatic fingerprint regions, indicating a greater degree of chemical reconstruction during the initial cycle. NPA undergoes the most severe changes: the strong pristine C═O band near ~1700 cm^−1^ and the broad carboxylic O─H region at 3000–2500 cm^−1^ decrease markedly upon lithiation and remain substantially altered after delithiation, while binder‐ and interphase‐related COO^−^ features become more dominant [29]. These FTIR results indicate that oxygen‐containing functional groups participate in interfacial evolution during the first electrochemical cycle, with partial recovery of the O─H‐related features occurring alongside persistent C─O/COO^−^‐related interphase reconstruction. DHN and especially NPA undergo more pronounced chemical evolution than the monohydroxylated derivatives.

The XPS survey spectra further support this interpretation (Figure 8g–h). Before cycling, the spectra were dominated primarily by C 1s and O 1s signals. After cycling, distinct F 1s, P 2p, and Li 1s peaks appear for all representative electrodes, indicating the formation of electrolyte‐derived interphase species containing fluorinated, phosphorus‐containing, and lithium‐containing components [30]. This behavior is consistent with the SEI formation from the decomposition of the LiPF_6_‐based electrolyte. More detailed information is obtained from the high‐resolution XPS spectra. In the F 1s region (Figure 9a–c), no obvious peak is observed before cycling, whereas after cycling, all representative electrodes show peaks assigned to LiF and residual LiPF_6_, indicating salt decomposition and fluoride‐containing interphase formation [31, 32, 33]. Similarly, no phosphorus signal is observed in the pristine P 2p spectra (Figure 9d–f), whereas after cycling, all materials display a dominant Li_*x*_PF_*y*_O*_z_* contribution together with a weaker LiPF_6_ signal, confirming the formation of fluorophosphate/phosphate species on the cycled surfaces [34, 35].

**FIGURE 9:**
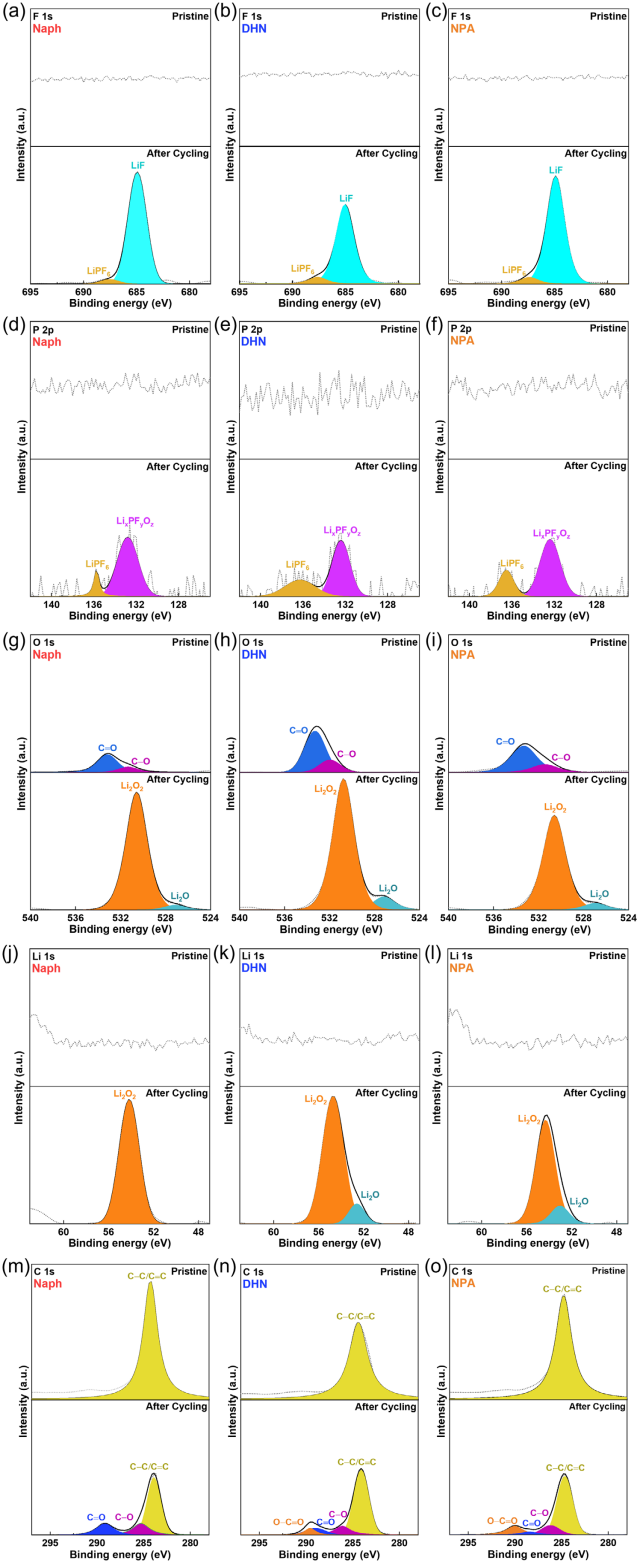
High‐resolution XPS analysis of representative electrodes before and after cycling. (a–c) F 1s, (d–f) P 2p, (g–i) O 1s, (j–l) Li 1s, and (m–o) C 1s spectra of Naph, DHN, and NPA electrodes.

More distinct differences among the derivatives are observed in the O 1s, Li 1s, and C 1s regions. Before cycling, all representative electrodes show C═O and C─O contributions in the O 1s spectra, with the overall oxygen‐related signal intensity following DHN > NPA > Naph (Figure 9g–i) [14, 15]. After cycling, the O 1s spectra show increased contributions from Li─O‐related inorganic interphase species [14, 33, 36]. The corresponding Li 1s spectra (Figure 9j–l) further reveal a more pronounced Li_2_O‐related contribution for DHN and NPA than for Naph, indicating a greater extent of reduced Li–O interfacial species in these oxygen‐functionalized electrodes. This trend is consistent with the DFT results, which show that DHN and NPA preferentially interact with Li/Li^+^ in their hydroxyl‐ and carboxyl‐containing regions, respectively. The stronger localized Li^+^ interactions at these oxygen‐containing sites may therefore facilitate more extensive Li‐associated interfacial reconstruction during cycling. In the C 1s spectra (Figure 9m–o), the pristine electrodes are mainly characterized by a C─C/C═C feature, while the cycled electrodes exhibit substantially more complex surface chemistry [14]. Naph remains comparatively simple, mainly showing C─C/C═C together with a C═O contribution [14, 37]. On the other hand, DHN and NPA display additional oxygenated carbon species, including C─O and O─C═O components [37]. The O─C═O signal is particularly pronounced for NPA and is absent in Naph, indicating more extensive organic/inorganic interphase buildup on the oxygen‐rich derivatives.

In summary, the EIS, FTIR, and XPS results establish that functional group chemistry governs not only dissolution behavior but also the extent of interfacial reconstruction during cycling. Naph and the monohydroxylated derivatives retain relatively stable surface chemistry and low interfacial resistance, whereas DHN and especially NPA undergo greater electrolyte‐induced dissolution, more extensive chemical reconstruction, and the formation of more resistive interphases. Combined with the dissolution and morphology results, these findings indicate that the specific oxygen‐containing functionalities in DHN and NPA are associated with greater active material loss and interfacial reconstruction, reducing electrochemically accessible material and increasing interfacial resistance.

## 4 Conclusions

This study establishes functional‐group chemistry as a decisive molecular parameter governing the dissolution behavior, interfacial stability, and electrochemical accessibility of naphthalene‐derived small‐molecule organic anodes. Through a systematic comparison of pristine naphthalene with hydroxyl‐ and carboxyl‐functionalized derivatives, we demonstrate that oxygen‐containing substitution does not simply modify the initial redox response but instead controls the coupled evolution of active material retention, interphase formation, and charge storage kinetics. Among the investigated materials, Naph and the monohydroxylated derivatives exhibit relatively stable electrochemical and interfacial behavior, whereas DHN and especially NPA show inferior performance due to significant active material loss and extensive interfacial reconstruction. These results identify dissolution‐induced material depletion and unstable interphase growth as the dominant degradation pathways in the more strongly oxygenated derivatives. More broadly, this work highlights that the performance of small‐molecule organic anodes is governed not only by intrinsic redox activity but also by how functional groups affect both Li/Li^+^ affinity and active material dissolution. The DFT results show that stronger local Li^+^ interactions do not necessarily improve electrochemical reversibility when accompanied by greater dissolution and interfacial reconstruction, ultimately reducing the fraction of electrochemically accessible material. These findings provide a clear molecular design guideline for developing stable organic electrode materials.

## Funding

This work was supported by the National Science and Technology Council, Taiwan (Grants 114‐2628‐E‐A49‐003 and 115‐2113‐M‐A49‐027‐MY2) and Ministry of Education, Taiwan (Grants MOE‐114‐YSFEE‐0010‐005‐P2 and Higher Education SPROUT Project at NYCU).

## Conflicts of Interest

The authors declare no conflicts of interest.

## Supporting information

Supplementary Material

## Data Availability

The data that support the findings of this study are available from the corresponding author upon reasonable request.
