# Supplementary material for: Functional Group‐Driven Dissolution and Interfacial Instability in Naphthalene‐Derived Organic Anodes for Li‐Ion Storage
Source: ChemSusChem. 2026 Nov 3;19(21):e71197. doi: 10.1002/cssc.71197 (PMC13729077; doi:10.1002/cssc.71197)
Supplement: Supplementary file 1 [file CSSC-19-e71197-s001.pdf]

Supporting Information

Functional Group-Driven Dissolution and Interfacial Instability in Naphthalene-Derived Organic Anodes for Li-ion Storage

Michael Silvano[1], Nguyet N. T. Pham[1], Duc-Anh Le[1] and Yu-Sheng Su*[1,2]*

[1]International College of Semiconductor Technology, National Yang Ming Chiao Tung University, 1001 Daxue Road, Hsinchu 300093, Taiwan

[2]Industry Academia Innovation School, National Yang Ming Chiao Tung University, 1001 Daxue Road, Hsinchu 300093, Taiwan

*Corresponding author: Yu-Sheng Su (yushengsu@nycu.edu.tw); Nguyet N. T. Pham (phamnn@nycu.edu.tw)

Table S1. Net mass changes of naphthalene-derived electrodes after cycling.

Molecules	Weight Change
Naphthalene	+30.8%
2,3-Dihydroxynaphthalene	−42.9%
1-Naphthol	+77.8%
2-Naphthol	+30%
2-Naphthoic Acid	−58.3%

Table S2. Extracted impedance parameters (R_e, R_{SEI}, and R_{ct}) of the naphthalene-derived electrodes at different cycling states.

Molecules	Cycling State	R_e (Ω)	R_{SEI} (Ω)	R_{ct} (Ω)
Naphthalene	Before cycle	5.4	-	84.1
	1st DChg	8.8	4.9	69.2
	1st Chg	9.2	3.6	117.8
	2nd DChg	14.0	12.8	61.6
	2nd Chg	15.7	7.5	101.4
	100th Chg	5.3	9.7	33.2
2,3-Dihydroxynaphthalene	Before cycle	50.8	-	469.2
	1st DChg	23.6	255.2	524.3
	1st Chg	6.6	331.2	48.3
	2nd DChg	9.6	128.7	583.3
	2nd Chg	27.4	249.0	288.9
	100th Chg	17.5	180.8	31.0
1-Naphthol	Before cycle	20.6	-	110.5
	1st DChg	6.5	46.3	248.2
	1st Chg	5.1	192.8	226.5
	2nd DChg	7.6	3.0	217.3
	2nd Chg	3.3	135.1	106.0
	100th Chg	10.9	6.3	60.1
2-Naphthol	Before cycle	9.6	-	77.3
	1st DChg	6.7	7.7	68.2
	1st Chg	18.1	76.2	169.9
	2nd DChg	2.6	12.4	134.2
	2nd Chg	9.0	65.7	119.7
	100th Chg	3.5	12.9	123.2
2-Naphthoic Acid	Before cycle	14.9	-	312.2
	1st DChg	7.0	80.0	30.3
	1st Chg	12.1	271.1	261.7
	2nd DChg	3.9	116.7	207.2
	2nd Chg	30.9	274.6	224.6
	100th Chg	8.9	281.9	510.3

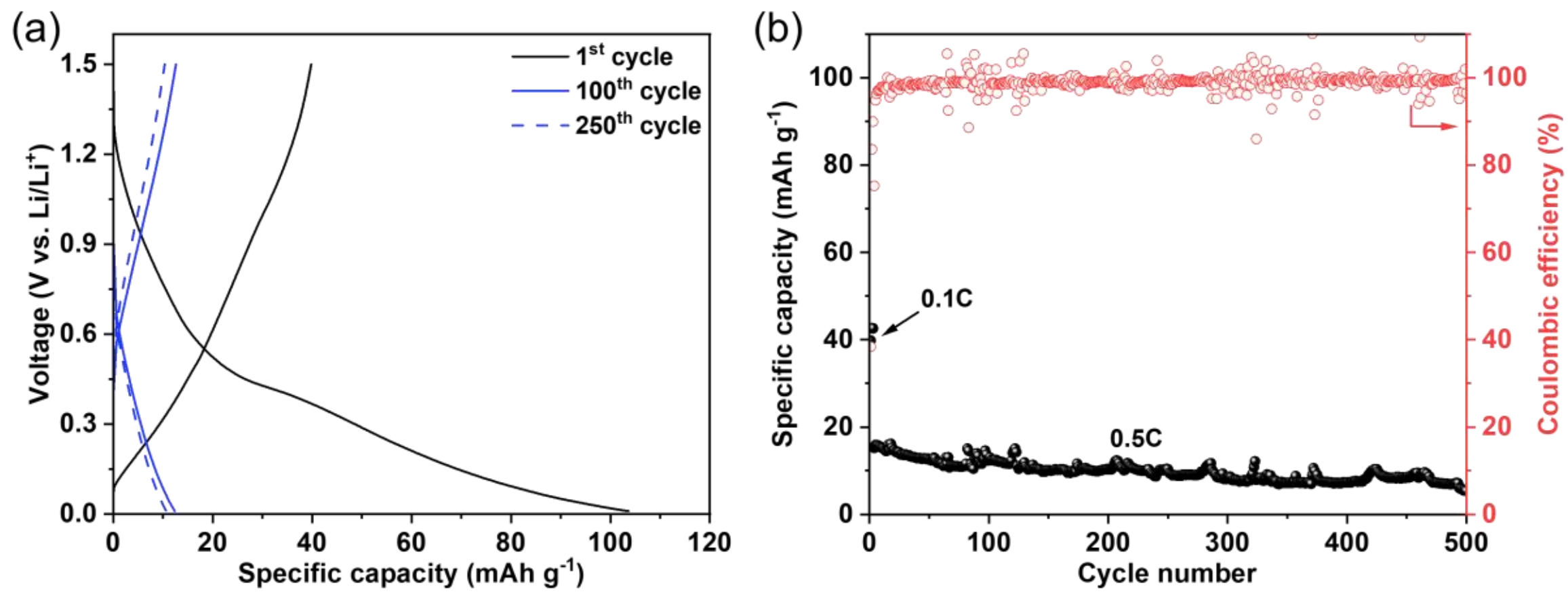

Figure S1. Electrochemical performance of the active material-free Super P/CMC/SBR control electrode. (a) Galvanostatic charge–discharge profiles at selected cycles and (b) cycling performance under the same electrochemical conditions used for the naphthalene-derived electrodes. Specific capacities are normalized to the mass of Super P.

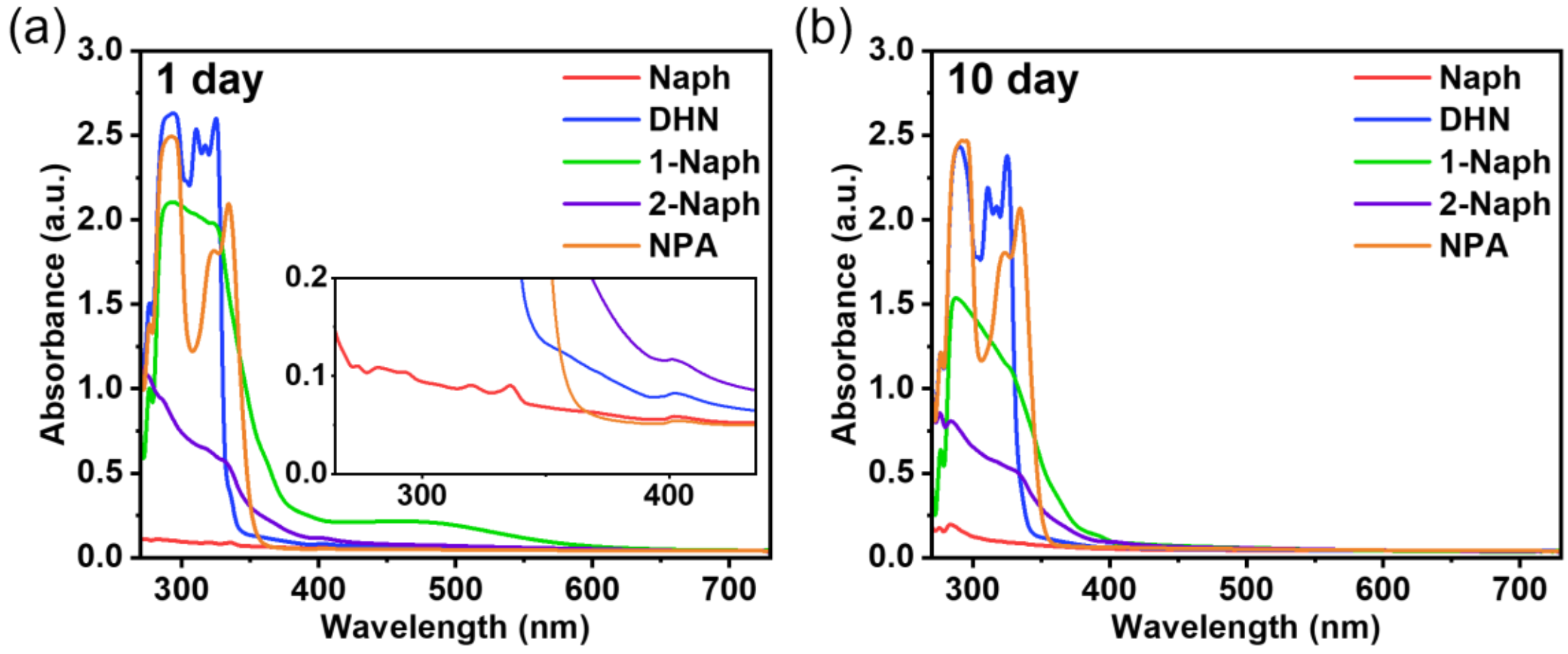

Figure S2. UV−Vis spectra obtained after static soaking of the naphthalene-derived molecular compounds in the carbonate-based electrolyte for (a) 1 day and (b) 10 days.
